# Supplementary material for: Predicting nuclear G-quadruplex RNA-binding proteins with roles in transcription and phase separation
Source: Nat Commun. 2024 Mar 22;15:2585. doi: 10.1038/s41467-024-46731-9 (PMC10959947; doi:10.1038/s41467-024-46731-9)
Supplement: Supplementary file 1 — Supplementary Information [file 41467_2024_46731_MOESM1_ESM.pdf]

Supplementary Materials

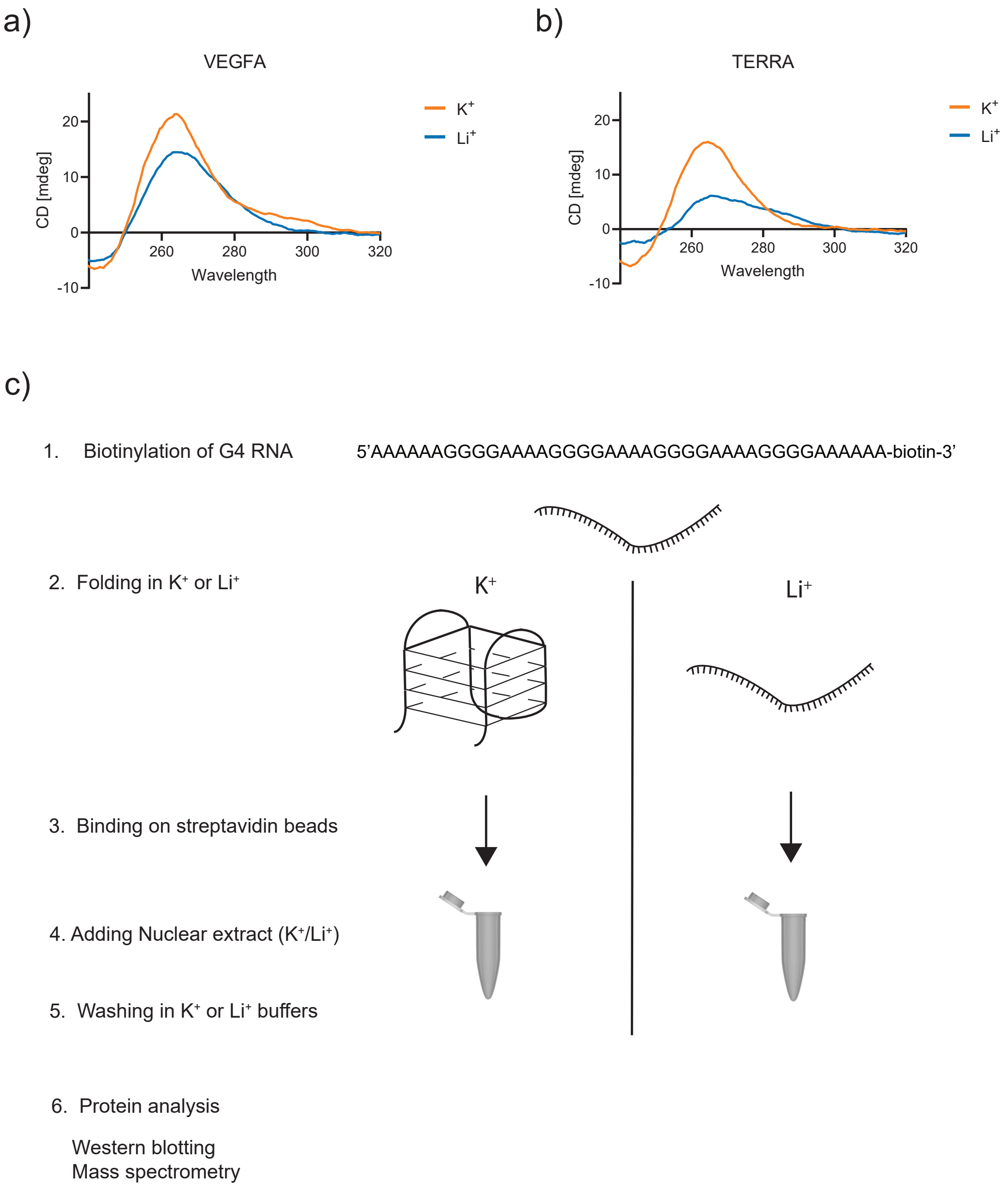
a)
VEGFA
CD [mdeg]
20
10
0
-10
260
280
300
320
Wavelength
K+
Li+
b)
TERRA
CD [mdeg]
20
10
0
-10
260
280
300
320
Wavelength
K+
Li+
c)
1. Biotinylation of G4 RNA
5'AAAAAAGGGGAAAAGGGGAAAAGGGGAAAAGGGGAAAAAA-biotin-3'
2. Folding in K+ or Li+
K+
Li+
3. Binding on streptavidin beads
4. Adding Nuclear extract (K+/Li+)
5. Washing in K+ or Li+ buffers
6. Protein analysis
Western blotting
Mass spectrometry

Supplementary Figure 1.

Circular dichroism spectrum for **a)** VEGFA and **b)** TERRA G4 forming oligonucleotides in K^+ and Li^+ buffer. **c)** An overview of the experimental design for purification of proteins binding to G-quadruplex forming RNA oligo.

a)

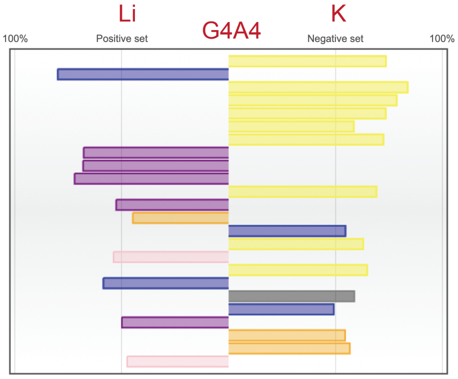

COV	AUC	Propensity	Z	P
73.65%	0.82	Nucleic Acid Binding - HB, Nucleic Acids Res. 2011, 39:D277-D282	24.5	<.00001
79.92%	0.81	Energy transfer from out to in(95%buried), Radzicka-Wolfenden, Biochemistry 1988, 27:1664-1670	17.0	<.00001
83.87%	0.88	Nucleic Acid Binding - nonclassicalRBD, Castello et al., Cell 2011, 149:1393-1406	15.2	<.00001
78.72%	0.86	Nucleic Acid Binding - interface_center, Terribilini et al., RNA 2006, 12:1450-1462	15.0	<.00001
73.61%	0.84	Nucleic Acid Binding - mRNAinteractome, Castello et al., Cell 2011, 149:1393-1406	13.4	<.00001
58.61%	0.73	Nucleic Acid Binding - HOH, Nucleic Acids Res. 2011, 39:D277-D282	13.2	<.00001
72.52%	0.81	Nucleic Acid Binding - interface, Nucleic Acids Res. 2011, 39:D277-D282	12.3	<.00001
67.78%	0.75	Hydrophobicity, Eisenberg et al, J. Mol. Biol. 1984, 179:125-142	10.1	<.00001
68.08%	0.77	Hydrophobicity, Abraham & Leo, Proteins: Structure; Func on and Gene cs 1987, 2:130-152	8.8	<.00001
72.11%	0.79	Hydrophobicity, Janin, Nature 1979, 277:491-492	8.8	<.00001
69.21%	0.79	Nucleic Acid Binding - unknownRBD, Castello et al., Cell 2011, 149:1393-1406	8.6	<.00001
52.56%	0.70	Hydrophobicity, Rao & Argos, Biochim. Biophys. Acta 1986, 869:197-214	8.4	<.00001
44.81%	0.64	Averaged turn propensi es in a transmembrane helix, Monne et al., J. Mol. Biol. 1999, 293:807-814	8.2	<.00001
54.70%	0.68	Mean volumes of residues buried in protein interiors, Harpaz et al., Structure 1994, 2:641-649	8.1	<.00001
63.01%	0.78	Nucleic Acid Binding - interface_close+1, Terribilini et al., RNA 2006, 12:1450-1462	7.9	<.00001
53.80%	0.67	Aggrega on high - Tartaglia, J Mol Biol 2008, 380(2):425-36	7.8	<.00001
64.89%	0.79	Nucleic Acid Binding - _interface_close-1, Terribilini et al., RNA 2006, 12:1450-1462	7.4	<.00001
58.61%	0.72	Percentage of buried residues, Janin et al., J. Mol. Biol. 1978, 125:357-386	7.3	<.00001
58.91%	0.71	B-Value / Dunker AK, Protein Pept Le . 2008; 15(9): 956-963	6.9	<.00001
49.21%	0.67	Average volume of buried residue, Chothia, Nature 1975, 254:304-308	6.6	<.00001
49.96%	0.68	Hydrophobicity, Kyte & Dooli le, J. Mol. Biol. 1982, 157:105-132	6.4	<.00001
54.51%	0.70	Turn propensity scale for transmembrane helices, Monne et al., J. Mol. Biol. 1999, 288:141-145	6.4	<.00001
56.73%	0.71	Knowledge-based membrane-propensity scale from 1D_Helix in MPtopo databases, Punta-Maritan, Proteins 2003, 50:114-121	6.2	<.00001
47.48%	0.66	Aggrega on med - Tartaglia, J Mol Biol 2008, 380(2):425-36	6.1	<.00001

b)

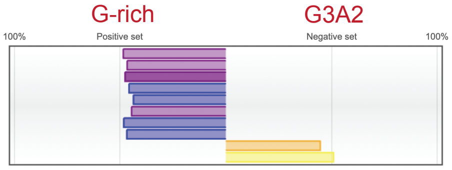

COV	AUC	Propensity	Z	P
48.51%	0.64	Hydrophobicity, Janin, Nature 1979, 277:491-492	8.7	<.00001
46.63%	0.64	Hydrophobicity, Eisenberg et al, J. Mol. Biol. 1984, 179:125-142	8.0	<.00001
47.60%	0.62	Hydrophobicity, Roseman, J. Mol. Biol. 1988, 200:513-522	7.8	<.00001
45.79%	0.63	Percentage of buried residues, Janin et al., J. Mol. Biol. 1978, 125:357-386	7.6	<.00001
43.66%	0.63	Propor on of residues 95% buried, Chothia, J. Mol. Biol. 1976, 105:1-14	6.4	<.00001
44.60%	0.63	Hydrophobicity, Abraham & Leo, Proteins: Structure; Func on and Gene cs 1987, 2:130-152	6.3	<.00001
48.26%	0.66	Propor on of residues 100% buried, Chothia, J. Mol. Biol. 1976, 105:1-14	6.0	<.00001
46.82%	0.65	Energy transfer from out to in(95%buried), Radzicka-Wolfenden, Biochemistry 1988, 27:1664-1670	5.7	<.00001
44.63%	0.60	Knowledge-based membrane-propensity scale from 1D_Helix in MPtopo databases, Punta-Maritan, Proteins 2003, 50:114-121	5.6	<.00001
50.93%	0.65	Nucleic Acid Binding - HB, Nucleic Acids Res. 2011, 39:D277-D282	5.4	<.00001

Supplementary Figure 2. Physico-Chemical Properties of **A**) G4A4 binding proteins in presence of K+ and Li+, **B**) G3A2 and G-rich proteins from literature [1.] **Left panels)** Each color represents a distinct property: yellow for RNA-binding ability, blue for burial, purple for hydrophobicity, pink for aggregation, grey for disorder and orange for membrane-binding. **Right panels)** The most effective discriminatory property within each category is highlighted. It is noteworthy that RNA-binding abilities are enriched in the G4A4 K+ and G3A2 binding groups, while hydrophobicity and burial are enriched in the G4A4 Li+ and G-rich binding groups. The amount of discriminated datasets (COV) and related Area under the ROC Curve (AUC), Z-score (Z) and P-values (P) of selected individual physico-chemical properties are reported [2].

a) Experimental Data

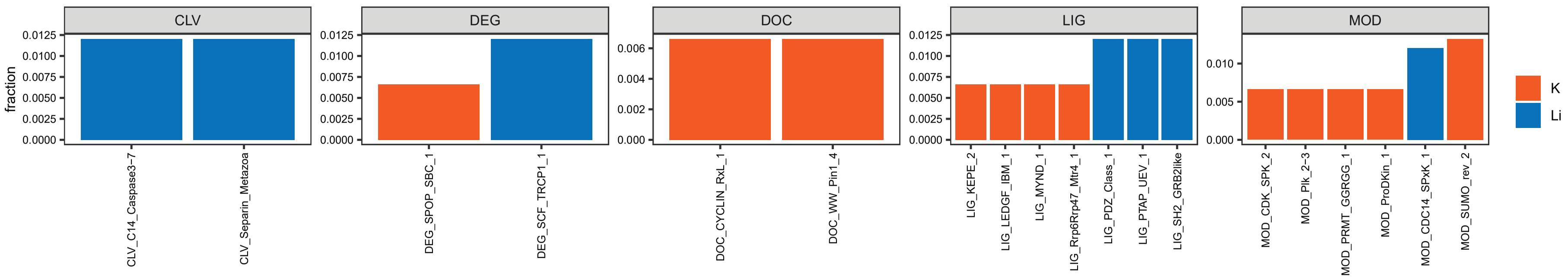

b) Predicted Data (top20 PTM per type)

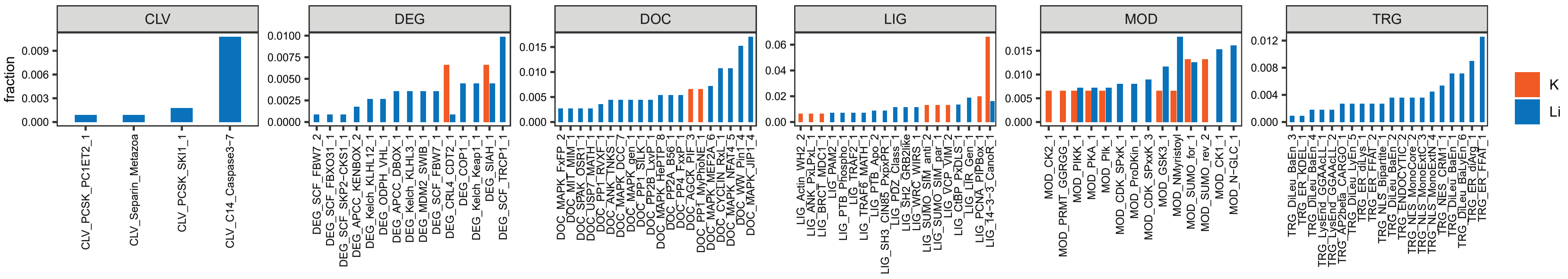

Supplementary Figure 3. A) Experimental (**top**) and **B**) predicted (**bottom**) PTMs annotations for Ligand (LIG), targeting (TRG), docking (DOC), degradation (DEG), modification (MOD), or cleavage (CLV) motifs for both proteins binding to G4A4 in the presence of K^+ or Li^+. The annotations are provided by ELM (http://elm.eu.org/). Red and blue bars represent the proteins in K^+ and Li^+ protein groups.

References (relative to the main text)

1. Herviou, P. *et al.* hnRNP H/F drive RNA G-quadruplex-mediated translation linked to genomic instability and therapy resistance in glioblastoma. *Nat Commun* **11**, 2661 (2020).
2. Klus, P. *et al.* The cleverSuite approach for protein characterization: predictions of structural properties, solubility, chaperone requirements and RNA-binding abilities. *Bioinformatics* **30**, 1601–1608 (2014).
