# Supplementary material for: Predicting nuclear G-quadruplex RNA-binding proteins with roles in transcription and phase separation
Source: Nat Commun. 2024 Mar 22;15:2585. doi: 10.1038/s41467-024-46731-9 (PMC10959947; doi:10.1038/s41467-024-46731-9)
Supplement: Supplementary file 2 — Description of Additional Supplementary Files [file 41467_2024_46731_MOESM2_ESM.pdf]

Supplementary Data 1.

*cat*RAPID predictions of chromatin related RBPs binding to folded or linear G4A4. **Column 1** contains gene name, **column** 2 corresponds to the Uniprot code, **columns 3** and **4** report the amino acids considered, **columns 5 and 6** are the *cat*RAPID scores for the folded and unfolded G4A4.

Supplementary Data 2.

Detected proteins in all conditions in mass spectrometry.

Supplementary Data 3.

Proteins detected and differentially bound in mass spectrometry analysis, including QUADRatlas status.

Supplementary Data 4.

Chromatin RBPs with prediction scores for binding to G4A4 in K^+ and Li^+ buffer.

Supplementary Data 5.

Secondary structure properties binding in presence of K^+ and Li^+ ions. **Column 1** is the AlphaFold name of the pdb. **Columns 2,3,4,5** report the amount of Coil, Turn, Helix and Strand per protein. **Column** 6 is the protein length and **column 7** the amount of structure normalized by length used in **Figure 4d.**

Supplementary Data 6.

Phase separation propensities computed with *cat*GRANULE for proteins binding to G4A4 in the presence of K^+ and Li^+.

Supplementary Data 7.

List of proteins reported in condensates (SG, PB and nucleolus).

Supplementary Data 8.

eCLIP set predictions with *clever*MACHINE scores 1) for G4 and 2) folded G4 propensities.

Supplementary Data 9

Chromatin set predictions with *clever*MACHINE scores 1) for G4 and 2) folded G4 propensities.

Supplementary Data 10.

Phase separation propensities computed with *cat*GRANULE for proteins predicted to bind G4A4 by G4-FUNNIES.
