# Supplementary material for: Predicting nuclear G-quadruplex RNA-binding proteins with roles in transcription and phase separation
Source: Nat Commun. 2024 Mar 22;15:2585. doi: 10.1038/s41467-024-46731-9 (PMC10959947; doi:10.1038/s41467-024-46731-9)
Supplement: Supplementary file 13 — Reporting Summary [file 41467_2024_46731_MOESM13_ESM.pdf]

nature portfolio

Corresponding author(s): Gian Gaetano Tartaglia
Ulf Andersson Vang Orom

Last updated by author(s): 2024-2-19

# Reporting Summary

Nature Portfolio wishes to improve the reproducibility of the work that we publish. This form provides structure for consistency and transparency in reporting. For further information on Nature Portfolio policies, see our Editorial Policies and the Editorial Policy Checklist.

## Statistics

For all statistical analyses, confirm that the following items are present in the figure legend, table legend, main text, or Methods section.

| n/a | Confirmed | |
|---|---|---|
| ☐ | ☒ | The exact sample size ($n$) for each experimental group/condition, given as a discrete number and unit of measurement |
| ☒ | ☐ | A statement on whether measurements were taken from distinct samples or whether the same sample was measured repeatedly |
| ☐ | ☒ | The statistical test(s) used AND whether they are one- or two-sided<br>*Only common tests should be described solely by name; describe more complex techniques in the Methods section.* |
| ☒ | ☐ | A description of all covariates tested |
| ☐ | ☒ | A description of any assumptions or corrections, such as tests of normality and adjustment for multiple comparisons |
| ☐ | ☒ | A full description of the statistical parameters including central tendency (e.g. means) or other basic estimates (e.g. regression coefficient) AND variation (e.g. standard deviation) or associated estimates of uncertainty (e.g. confidence intervals) |
| ☐ | ☒ | For null hypothesis testing, the test statistic (e.g. $F$, $t$, $r$) with confidence intervals, effect sizes, degrees of freedom and $P$ value noted<br>*Give P values as exact values whenever suitable.* |
| ☒ | ☐ | For Bayesian analysis, information on the choice of priors and Markov chain Monte Carlo settings |
| ☒ | ☐ | For hierarchical and complex designs, identification of the appropriate level for tests and full reporting of outcomes |
| ☒ | ☐ | Estimates of effect sizes (e.g. Cohen's $d$, Pearson's $r$), indicating how they were calculated |

*Our web collection on statistics for biologists contains articles on many of the points above.*

## Software and code

Policy information about availability of computer code

| | |
|---|---|
| Data collection | No software was used for data collection. |
| Data analysis | The webservice presented in the manuscript http://service.tartaglialab.com/new_submission/G4FUNNIES is an application of a general algorithm that we previously published https://pubmed.ncbi.nlm.nih.gov/24493033/.<br><br>To predict the G4 binding ability of proteins we used the catRAPID algorithm with the default parameters reported in https://pubmed.ncbi.nlm.nih.gov/21623348/. |

For manuscripts utilizing custom algorithms or software that are central to the research but not yet described in published literature, software must be made available to editors and reviewers. We strongly encourage code deposition in a community repository (e.g. GitHub). See the Nature Portfolio guidelines for submitting code & software for further information.

## Data

Policy information about availability of data

All manuscripts must include a data availability statement. This statement should provide the following information, where applicable:

- Accession codes, unique identifiers, or web links for publicly available datasets
- A description of any restrictions on data availability
- For clinical datasets or third party data, please ensure that the statement adheres to our policy

Source data are provided with this paper. The mass spectrometry proteomics data generated in this study have been deposited to the ProteomeXchange Consortium via PRIDE repository https://www.ebi.ac.uk/pride/archive with dataset identifier PXD041154.
The sequences utilized in the manuscript were obtained from Uniprot, accessible at https://www.uniprot.org/, and their respective structures were sourced from AlphaFold, available at https://alphafold.ebi.ac.uk/. The catalog of phase-separating proteins was taken from https://llps.biocuckoo.cn/index.php.

## Research involving human participants, their data, or biological material

Policy information about studies with human participants or human data. See also policy information about sex, gender (identity/presentation), and sexual orientation and race, ethnicity and racism.

| | |
|---|---|
| Reporting on sex and gender | N/A |
| Reporting on race, ethnicity, or other socially relevant groupings | N/A |
| Population characteristics | N/A |
| Recruitment | N/A |
| Ethics oversight | N/A |

Note that full information on the approval of the study protocol must also be provided in the manuscript.

# Field-specific reporting

Please select the one below that is the best fit for your research. If you are not sure, read the appropriate sections before making your selection.

☒ Life sciences ☐ Behavioural & social sciences ☐ Ecological, evolutionary & environmental sciences

For a reference copy of the document with all sections, see nature.com/documents/nr-reporting-summary-flat.pdf

# Life sciences study design

All studies must disclose on these points even when the disclosure is negative.

| | |
|---|---|
| Sample size | The whole cellular proteome was analyzed by mass-spectrometry and the sample size was determined by the enrichments of K+ and Li+ protein groups. |
| Data exclusions | No data were excluded |
| Replication | All the experiments were replicated 1) technically and 2) biologically.Technical replication involves repeating the experiments using the same methods, conditions, and equipment to verify the precision and reliability of the initial results. Biological replication involves repeating the experiments on different biological samples that are independent of the original set. |
| Randomization | No randomization, as the work is based on two contrasting categories (K+ and Li+) groups |
| Blinding | N/A |

# Reporting for specific materials, systems and methods

We require information from authors about some types of materials, experimental systems and methods used in many studies. Here, indicate whether each material, system or method listed is relevant to your study. If you are not sure if a list item applies to your research, read the appropriate section before selecting a response.

## Materials & experimental systems

| n/a | Involved in the study |
| --- | --- |
| ☐ | ☒ Antibodies |
| ☐ | ☒ Eukaryotic cell lines |
| ☒ | ☐ Palaeontology and archaeology |
| ☒ | ☐ Animals and other organisms |
| ☒ | ☐ Clinical data |
| ☒ | ☐ Dual use research of concern |
| ☒ | ☐ Plants |

## Methods

| n/a | Involved in the study |
| --- | --- |
| ☒ | ☐ ChIP-seq |
| ☒ | ☐ Flow cytometry |
| ☐ | ☐ MRI-based neuroimaging |

# Antibodies

Antibodies used

Ruvbl2 (Abcam ab36569)

NCL (Abcam, ab136649)

Goat anti-mouse (Thermo Fischer Scientific, G-21040)

Goat anti-rabbit (Thermo Fischer Scientific, 31460)

Validation

The antibodies were commercially validated:

https://www.abcam.com/en-it/products/primary-antibodies/reptin-tip49b-ruvb2-antibody-ab36569#
https://www.abcam.com/en-it/products/primary-antibodies/nucleolin-antibody-364-5-ab136649
https://www.thermofisher.com/antibody/product/Goat-anti-Mouse-IgG-H-L-Cross-Adsorbed-Secondary-Antibody-Polyclonal/G-21040
https://www.thermofisher.com/antibody/product/Goat-anti-Rabbit-IgG-H-L-Secondary-Antibody-Polyclonal/31460

About their use:

For western blot validation, elution of proteins was carried out by incubating the beads at 80 °C with 2.5X NuPAGE LDS sample buffer (NP0007, Thermo Fisher Scientific). Samples were loaded on 4-12% NuPAGE Bis-Tris polyacrylamide gel (NP0322, Thermo Fischer Scientific), running with NuPAGE MOPS SDS running buffer (NP0001, Thermo Fischer Scientific) at 110 V for 120 minutes. Transfer to Immobilon-P 0.45µm PVDF membrane (Merck Millipore) was carried out in NuPAGE Transfer buffer (NP00061, Thermo Fischer Scientific), supplemented with 10% methanol. Transfer conditions were 120 minutes with constant voltage at 100 V. Blots were blocked with 5% skim milk solution in PBS-T [1X PBS, 0.05% Tween-20]. Incubations with primary antibodies were carried out overnight as a 1:1,000 dilution. Antibody incubations were followed by washing with PBS-T 3 times for 5-10 minutes. Secondary antibody against rabbit/mouse IgG was diluted 1:10,000 in PBS-T. Signal was detected with the SuperSignal ECL reagent (34579, Thermo Fischer Scientific) and visualized with GE Amersham Imager 600. Western blot bands were quantified with Fiji software (Schindelin et al., 2012). Quantified band intensities from pulldowns were normalized to protein levels in input lysates and expressed as pulldown/nuclear extract ratio. Three or four replicate experiments were used for quantification, statistical significance was estimated with paired Student's t-test (*p<0,05).

Native RIP for endogenous mRNAs (VEGFA, MYC, BCL-2, NRAS) and non-coding RNAs (RN7SK, GAS5) was carried out using Ruvbl2 and Rabbit IgG1 antibodies. Protein A and G coated Dynabeads (Thermo Fisher) were first washed in RIP lysis buffer, containing 25 mM TRIS, 150 mM KCl, 0.5% Igepal CA-630, 5 mM DTT, 20U/ml Rnase inhibitor (Rnasin, Promega), 1X Protease inhibitor cocktail (cOmplete, Roche). Prior to immunoprecipitation, antibodies were incubated with Protein A/G beads, using 4.8 µg of antibodies with 20 µl A+G beads. 20-30 million K562 were harvested for each RNA immunoprecipitation and lysed with 700 ul RIP lysis buffer for 25 min on ice, after which lysates were centrifuged 25 min at 4 degrees x 17,000g. 1% of lysate was removed for input analysis. RNA immunoprecipitation was carried out over 16 h at 4 degrees with rotation. Beads were washed 5 times 10 minutes. Protein-bound RNA was eluted by incubating the beads with TRIzol, following RNA extraction. cDNA was prepared from equal volumes of immunoprecipitated and input RNA using Maxima H Minus reverse transcriptase (Thermo Fisher) and random hexamer primers. cDNA was diluted for detection of RN7SK transcript 1:130. qPCR analysis was carried out using Platinum SYBR Green (Thermo Fisher). CT values were converted by 2^^-CT method and normalized to input levels. Statistical significance was estimated using two-way ANOVA, with Šídák's multiple comparisons test (GraphPad Prism). Statistical significance levels based on p-value: ns = not significant; *p <0.05; **p <0.01; ***p <0.001.

# Eukaryotic cell lines

Policy information about cell lines and Sex and Gender in Research

| | |
| --- | --- |
| Cell line source(s) | K562 human myelogenous leukemia cell line (ATCC CCL-243) was obtained from ATCC. |
| Authentication | Obtained from ATCC (ATCC CCL-243), as https://www.atcc.org/products/ccl-243. |
| Mycoplasma contamination | Cell lines routinely tested negative for mycoplasm. |

| | |
|---|---|
| Commonly misidentified lines (See ICLAC register) | No commonly misidentified cell line was used in the study. |

## Plants

| | |
|---|---|
| Seed stocks | N/A |
| Novel plant genotypes | N/A |
| Authentication | N/A |

## Magnetic resonance imaging

### Experimental design

| | |
|---|---|
| Design type | N/A |
| Design specifications | N/A |
| Behavioral performance measures | N/A |

### Acquisition

| | |
|---|---|
| Imaging type(s) | N/A |
| Field strength | N/A |
| Sequence & imaging parameters | N/A |
| Area of acquisition | *State whether a whole brain scan was used OR define the area of acquisition, describing how the region was determined.* |

Diffusion MRI ☐ Used ☐ Not used

### Preprocessing

| | |
|---|---|
| Preprocessing software | N/A |
| Normalization | N/A |
| Normalization template | N/A |
| Noise and artifact removal | N/A |
| Volume censoring | N/A |

### Statistical modeling & inference

| | |
|---|---|
| Model type and settings | N/A |
| Effect(s) tested | N/A |

Specify type of analysis: ☐ Whole brain ☐ ROI-based ☐ Both

| | |
|---|---|
| Statistic type for inference (See Eklund et al. 2016) | N/A |
| Correction | N/A |

## Models & analysis

| n/a | Involved in the study |
| --- | --- |
| ☐ | ☐ Functional and/or effective connectivity |
| ☐ | ☐ Graph analysis |
| ☐ | ☐ Multivariate modeling or predictive analysis |

| | |
| --- | --- |
| Functional and/or effective connectivity | N/A |
| Graph analysis | N/A |
| Multivariate modeling and predictive analysis | N/A |
